# Maternal Reproductive Toxicity of Some Essential Oils and Their Constituents

**DOI:** 10.3390/ijms22052380

**Published:** 2021-02-27

**Authors:** Noura S. Dosoky, William N. Setzer

**Affiliations:** 1 Aromatic Plant Research Center, Lehi, UT 84043, USA; 2 Department of Chemistry, University of Alabama in Huntsville, Huntsville, AL 35899, USA; wsetzer@chemistry.uah.edu

**Keywords:** essential oils, pregnancy, anethole, *trans*-sabinyl acetate, camphor, methyl salicylate, thujone, pulegone, citral, apiole

## Abstract

Even though several plants can improve the female reproductive function, the use of herbs, herbal preparations, or essential oils during pregnancy is questionable. This review is focused on the effects of some essential oils and their constituents on the female reproductive system during pregnancy and on the development of the fetus. The major concerns include causing abortion, reproductive hormone modulation, maternal toxicity, teratogenicity, and embryo-fetotoxicity. This work summarizes the important studies on the reproductive effects of essential oil constituents anethole, apiole, citral, camphor, thymoquinone, *trans*-sabinyl acetate, methyl salicylate, thujone, pulegone, β-elemene, β-eudesmol, and costus lactone, among others.

## 1. Introduction

The female reproductive cycle involves a very complex sequence of changes in the uterus, ovaries, breasts, and regulatory hormone levels. Several mechanisms, metabolic pathways, and enzymes are involved in controlling and regulating reproductive hormone levels in the blood. During the reproductive cycle, these endogenous hormones are responsible for preparing for implantation and for milk production [1,2]. Out of concern of adversely affecting the unborn child, some pregnant women prefer to use herbs, herbal preparations, or oils rather than conventional medication to treat pregnancy-related symptoms (morning sickness, nausea, vomiting, heartburn, etc.) [3,4]. Indeed, several plants can improve the female reproductive function and some are beneficial during pregnancy, childbirth, and postpartum [5,6]. Similarly, essential oils (EOs) are generally safe, and many oils have a generally recognized as safe (GRAS) status. However, the use of herbs and EOs during pregnancy is a highly controversial matter. It is worth mentioning that it is the individual composition of an EO and the possible hazardousness of a single or a group of constituents that determine their medical and therapeutic usage. Some EO-containing plant species are highly variable and may produce several EO chemotypes with different EO compositions of which one or some chemotypes possess potential maternal reproductive toxicity. Causing abortion is a major concern. It is generally believed that EOs extracted from emmenagogic plants are dangerous or unsafe in pregnancy, as they might cause menstrual bleeding and lead to miscarriage. Yet, that is not always true. The oils do not necessarily carry the same activity as the whole plant. Regardless of their ability to promote menstruation, there is no decisive evidence that these oils are abortifacient in aromatherapy amounts. For instance, the whole plants of savin, pennyroyal, tansy, and rue can induce miscarriage and their oils were on the list of abortifacient oils at some point [7]. Thus far, these oils showed no activity on uterine muscle of isolated human uterus [8] and did not cause fetal death [9,10]. Still, these facts do not prove the safety of these oils. Since the toxicity of many plants and EOs has not been studied yet, the concern cannot be completely dismissed.

Another major concern about essential oils and their constituents is mimicking, interfering with, or antagonizing the action of reproductive hormones, which in turn could disrupt the reproductive and developmental processes [11]. Some EO constituents could affect the outcome of pregnancy through causing maternal toxicity, teratogenicity, embryo-fetotoxicity, or anti-angiogenicity [12]. Since angiogenesis is essential for a successful pregnancy, anti-angiogenic oil constituents carry the risk of causing preeclampsia, growth restriction, and fetal death [13,14]. Moreover, at high doses, teratogenic oils or components can cause birth defects of a structural nature that arise during embryonic development [15]. Generally, EO components can cross the placenta to the fetal circulation due to their low molecular weight, protein binding ability, and lipophilicity and can cause fetotoxicity [16]. Components that cross the placenta are more likely to affect the fetal central nervous system (CNS) since it is underdeveloped [17]. Unsaturated compounds like cinnamaldehyde, citral, and β-pulegone act via interacting with the lipids in embryo cell membranes [18]. Subcutaneous injection of 1,8-cineole (at 500 mg/kg for four days) to pregnant rats affected the fetal liver enzymes activity [19]. Similar to crossing the placenta, most flavor and EO constituents are expected to pass into breast milk via passive diffusion [20]. In lactating mice, constituents of sandalwood oil were able to pass to maternal milk and affected infant hepatic metabolic enzymes [21].

**Figure 1:**
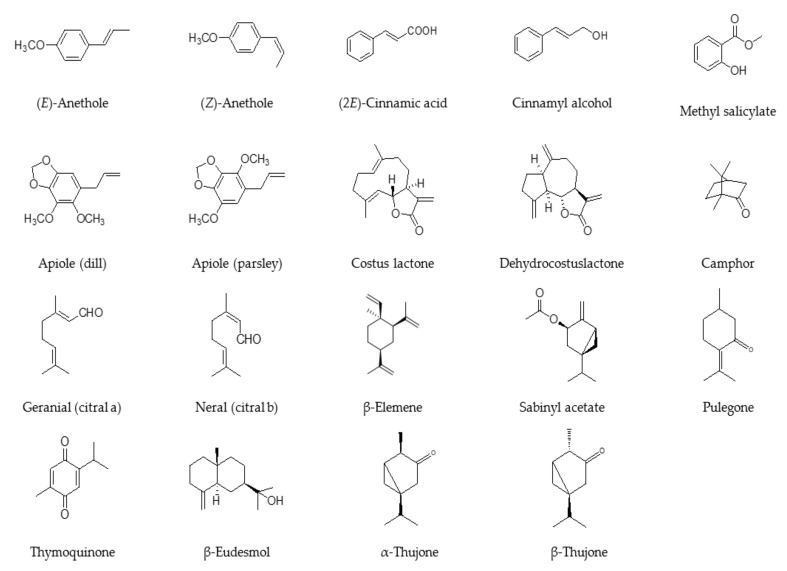
Chemical structures of key essential oil components discussed in this review.

This review has been prepared based on a comprehensive survey of major scientific databases for the effects of some EOs and their constituents on the female reproductive system during pregnancy and on the development of the fetus. Figure 1 presents the chemical structures of key essential oil components discussed in this review, while the chemical composition of these oils is summarized in Table 1. It should be noted that because of the ethical and safety issues in human toxicity testing, a substantial weight is usually given to animal testing for reproductive toxicity despite the major differences in reproductive physiologies. EOs or components that showed animal reproductive toxicity should either be prohibited (strictly avoided) or restricted (used with some degree of caution) during pregnancy and lactation.

## 2. Anethole-Rich Essential Oils

(*E*)-Anethole is a phenylpropenoid ether, found in anise (*Pimpinella anisum* L.) oil (75.2–96.1%) [23,24], aniseed myrtle (*Syzygium anisatum* (Vickery) Craven and Biffin) oil (95.0%) [25], sweet fennel (*Foeniculum vulgare* Mill.) oil (58.1–92.5%) [23,24], star anise (*Illicium verum* Hook. f.) oil (71.2–91.8%) [23], bitter fennel (*Foeniculum vulgare* Mill. subsp. *capillaceum* Gilib.) oil (52.5–84.3%) [23], and betel oil (*Piper betle* L.) (0–7.8%) [22]. (*Z*)-Anethole, an isomer of (*E*)-anethole, is found in sweet fennel oil (tr–0.7%), anise (tr–0.5%), star anise (tr–0.4%), and bitter fennel (tr–0.2%) [22]. (*Z*)-Anethole is considerably more toxic than the common isomer (*E*)-anethole [74].

(*E*)-Anethole was reported to have anti-hypernociceptive, anticancer, antiplatelet, anti-inflammatory, and anesthetic properties [75,76,77]. However, administration of (*E*)-anethole-rich EOs (by any route) should be avoided in pregnancy, breastfeeding, and estrogen-dependent cancers. Additionally, internal use of (*E*)-anethole-rich EOs is not advisable in childbirth due to its antiplatelet aggregation activity. There is enough evidence for the estrogenic action of (*E*)-anethole. (*E*)-Anethole was estrogenic in yeast assays (in vitro) [1,78]. It was reported to bind to estrogen receptors in engineered yeast cells [1]. In humans, sweet fennel tea (rich in (*E*)-anethole) was estrogenic in vivo [79]. A notable increase in uterine weight was observed in immature female rats following (*E*)-anethole treatment (80 mg/kg/day for 3 days), confirming its estrogenic effect [80]. (*E*)-Anethole showed an anti-implantation effect in pregnant rats. Oral administration of (*E*)-anethole (50, 70, and 80 mg/kg on gestational days 1–10) to pregnant albino Charles Foster rats caused a dose-dependent reduction in implantation as a result of a disruption of hormonal balance [80]. It is worth mentioning that both mice and humans can metabolize (*E*)-anethole in a similar way, while rats metabolize it differently [81]. A metabolite of anethole, anethole-1’,2’-epoxide, was carcinogenic and caused the formation of hepatomas and papillomas in mice [82].

Anethole-rich essential oils such as aniseed, star anise, bitter fennel, sweet fennel, and aniseed myrtle are estrogenic in one or more in vitro assays and may cause reproductive hormone modulation [1,78]. These oils are hepatotoxic due to their high (*E*)-anethole content. Bitter fennel oil is hepatotoxic due to the metabolite, anethole-1’,2’-epoxide [83]. Like bitter fennel EO, sweet fennel EO is a reproductive hormonal level modulator, fetotoxic, and hepatotoxic [84]. Sweet fennel tea (containing 1.3–10.0% of the oil [85]) showed in vivo estrogenic activity in humans and its prolonged use caused premature breast development and significantly higher serum estradiol levels [79]. A sweet fennel oil (with 72% (*E*)-anethole, 12.0% fenchone, and 5% estragole) was teratogenic at 0.93 mg/mL and produced about 50% reduction in differentiated rat embryo limb bud foci. It dose-dependently decreased the intensity of oxytocin or prostaglandin E2-induced uterine contractions ex vivo [86] which is why the use of sweet fennel oil is not advisable during slow-progressing labor. Therefore, consumption of anethole-rich essential oils is unsafe and should be avoided (by any route) during pregnancy, breastfeeding, and in some estrogen-dependent cancers [87,88]. These oils are potentially carcinogenic based on their estragole and safrole (minor components) content [89]. (*E*)-Anethole and estragole interfered with fetoplacental steroidogenesis in a co-culture of human adrenocortical carcinoma cells (H295R) and human placental choriocarcinoma cells (BeWo) cells by increasing hormonal concentrations and altering steroidogenic enzyme activity and expression [90,91].

## 3. Methyl Salicylate-Rich Essential Oils

Methyl salicylate is a phenolic ester that dominates wintergreen (*Gaultheria procumbens* L.) (96.0–99.5%) [92] and sweet birch (*Betula lenta* L.) (90.4%) oils [22]. Methyl salicylate is largely hydrolyzed into salicylic acid in the liver [93]. Following topical application in humans, methyl salicylate can be transdermally absorbed and converted to salicylic acid in the dermal and subcutaneous tissues [94,95]. Orally taken methyl salicylate is metabolized faster in rats and dogs than in humans which means a higher toxicity in humans [96]. Methyl salicylate poisoning in humans is known to cause fever, nausea, vomiting, CNS excitation, tachycardia, rapid breathing, high blood pressure, respiratory failure, pneumonia, pulmonary edema, convulsions, and coma [97]. Methyl salicylate poisoning in humans has a 50–60% mortality rate which is a result of cardiovascular collapse and respiratory failure [98,99]. Methyl salicylate showed in vitro human estrogen receptor α (hERα) agonistic activity [100]. Salicylates have been shown to cross the placenta [101] and lead to restricted growth and congenital abnormalities in animal experiments [102]. The fact that superoxide dismutase treatment prevented salicylate-induced malformations in rat embryos suggests that free oxygen radicals play a role in its teratogenic action [103]. Intraperitoneal injection of methyl salicylate (200 or 400 mg/kg, pregnant rats on gestational days 9 and 10) dose-dependently reduced the development of the brain, lung, liver, and kidney of the fetus [104]. Methyl salicylate (i.p., 50 or 100 µL) given to female rats on gestational days 10 and 11 resulted in retardation of fetal kidney development. At 100 µL, maternal weight gain was retarded, the offspring were fewer and smaller, and resorptions and malformations were increased [105]. A single subcutaneous injection of methyl salicylate (1.5 mL/kg on gestation day 7, 9, or 11) to female rats resulted in higher fetal deaths, reduced fetal weight, and cleft palate and tail abnormalities [106]. At 200, 250, or 300 mg/kg/day (i.p., on gestation days 11–12), methyl salicylate showed teratogenicity and embryotoxicity in pregnant Sprague-Dawley rats [107]. Methyl salicylate increases the occurrence of dilated renal pelvis in the rat fetus and causes a temporary maturation delay in the rat’s ability to concentrate urine [107]. In another experiment, several anomalies were observed in the offspring digestive tract, CNS, liver, and skeleton following a single subcutaneous injection of methyl salicylate (0.1–0.5 mL on gestational day 9, 10, or 11) in pregnant rats [108]. Subcutaneous administration of methyl salicylate (400 mg/kg) caused a substantial decrease in plasma calcium levels in pregnant rats and mice which might be linked to the fetal toxicity [109]. In pregnant hamsters, oral or topical methyl salicylate caused neural tube fusion failure in the embryos [93,110]. In addition, high oral doses of wintergreen EO were toxic and teratogenic in rats and monkeys [102]. Based on the available information, use of methyl salicylate-rich essential oils or any preparations containing them, by any route, should be avoided during pregnancy and lactation.

## 4. *cis*-Sabinyl Acetate-Rich Essential Oils

*cis*-Sabinyl acetate is a bicyclic monoterpenoid ester found in *Plectranthus* (*P. fruticosus* L’Hér.) (>60.0%) [59], savin (*Juniperus sabina* L.) (19.1–53.1%) [32], wormwood (*Artemisia absinthium* L., β-thujone chemotype) (18.1–32.8%) [32,41,69], wormwood (sabinyl acetate chemotype) (31.5%) [32,39], wormwood ((*Z*)-epoxy ocimene chemotype) (0.3–7.4%) [32,39], and Spanish sage (*Salvia lavandulifolia* Vahl) (0–6.6%) [32,65]. It is considered one of the very few toxic essential oil esters. Sabinyl acetate is among the most dangerous constituents in pregnancy since it is maternally toxic and abortifacient [22]. It acts through inhibiting implantation of the embryo [111]. Experiments with sabinyl acetate-rich oils suggest substantial reproductive toxicity risks. *Plectranthus* oil (60% sabinyl acetate) is embryotoxic, fetotoxic, teratogenic, and abortifacient [112]. In pregnant rats, oral administration of *Plectranthus* oil at 0.5, 2.5, or 5.0 mg/kg on gestational days 6–15 caused a surge in the rate of resorption and fetal toxicity (abnormally small eyeballs and lack of eyes) [113]. At a dose of 5 mg/kg, *Plectranthus* oil showed abortifacient and fetotoxic effects in pregnant rats [59]. The strong embryotoxic and fetotoxic action of *Plectranthus* oil was attributed to its sabinyl acetate content [113]. Subcutaneously injected *Plectranthus* EO (15, 45, or 135 mg/kg) to pregnant mice on gestational days 6–15 caused abortion, malformed embryos (kidney and heart defects, skeletal modifications, and lack of eyes), and a rise in resorption in mice [114,115]. Extracts of *Plectranthus fruticosus* showed antifertility and anti-implantation effects in Wistar rats [112].

Another sabinyl acetate-rich oil is savin oil (*Juniperus sabina* L.). Savin oil (50% sabinyl acetate) is embryo-fetotoxic, abortifacient, and hepatotoxic [116]. It can easily cross the placenta and cause abortion [117]. Subcutaneous administration of savin EO to pregnant mice (at 15, 45, or 135 mg/kg on gestational days 6–15) caused embryotoxicity and significant weight loss [116]. It also inhibited implantation in mice on gestational days 0–4 but not on gestational days 8–11 suggesting that sabinyl acetate causes abortion [111]. The abortifacient action of the savin plant does not seem to be only due to the oil. An ether extract of *Juniperus sabina*, prepared after isolating the oil, showed anti-implantation effect in a dose-dependent manner [118]. Since nothing much can be gained from using savin oil, it should not be used either internally or externally. Similarly, juniper berry (*Juniperus communis* L.) ethanolic extract was clearly abortifacient [119]; however, there is no evidence that the juniper berry EO is abortifacient.

Moreover, Spanish sage oil is a well-known abortifacient. Subcutaneously injected Spanish sage oil fraction (50% sabinyl acetate) into pregnant mice (at 15, 45, and 135 mg/kg on gestational days 6–15) caused abortion and maternal toxicity in a dose-dependent manner [115]. Spanish sage oil (0.01 mg/mL) also induced β-galactosidase activity in yeast which suggests a possible estrogenic activity [120]. Similarly, wormwood oil is neurotoxic, embryo-fetotoxic, and abortifacient [23,39]. It is particularly hazardous since it carries combined risks from thujones and sabinyl acetate. Since there is no established no observed adverse effect level (NOAEL), it is best to completely avoid sabinyl acetate-rich essential oils and any preparations containing them in pregnancy, especially during the first trimester.

## 5. Thujone-Rich Essential Oils

Thujone is a bicyclic monoterpenoid ketone present as two isomers found together in essential oils: (*S*)-α-thujone and (*R*)-β-thujone. (S)-α-Thujone is a major component in western red cedar (*Thuja plicata* Donn ex D. Don) (63.5–84.0%) [68], génépi (*Artemisia genipi* Weber ex Stechm.) (79.8%) [38], sea wormwood (*Artemisia maritima* L.) (63.3%) [63], thuja (*Thuja occidentalis* L.) (48.7–51.5%) [121,122,123], Dalmatian sage (*Salvia officinalis* L.) (13.1–48.5%) [36], white wormwood (*Artemisia herba-alba* Asso) (25.7–36.8%) [23,41,69], lanyana (*Artemisia afra* Jacq. ex Willd.) (22.5%) [22], and common mugwort (*Artemisia vulgaris* L., camphor/thujone chemotype) (11.4%) [49]. (*R*)-β-Thujone is found in wormwood (β-thujone chemotype) (33.1–59.9%) [23,41,69], tansy (*Tanacetum vulgare* L.) (45.2%) [117], great mugwort (*Artemisia arborescens* (Vaill.) L.) (34.0%) [39], wormwood (β-thujone/(Z)-epoxyocimene chemotype) (20.9–21.7%), Dalmatian sage (3.9–19.1%) [36], western red cedar (4.9–15.2%) [68], génépi (10.4%) [38], thuja (3.14 –9.9%) [121,122,123], white wormwood (2.0–9.0%) [23,41,69], and lanyana (8.9%) [22]. α-Thujone is more neurotoxic than β-thujone. When taken orally, thujone can affect the CNS and cause convulsions which suggests that it can cross the blood–brain barrier [124,125]. Even at low doses, thujone can affect nervous tissue in rats [126]. Thujone diastereomers have been shown to inhibit human gamma-aminobutyric acid type A (GABA_A_) receptor currents [127,128] which is the mechanism behind causing muscle spasms and convulsions [129,130]. Thujone has a NOAEL for convulsions of 5 mg/kg in female rats [131]. CYP2A6, followed by CYP3A4 and CYP2B6, metabolizes α-thujone to 4- and 7-hydroxythujone in humans [130]. α-Thujone was found to inhibit CYP2A6 (IC_50_ = 2.34 mg/L) and CYP2B6 (IC_50_ = 2.66 mg/L) which could contribute to a lengthy and amplified α-thujone toxicity [132]. Dalmatian sage EO causes convulsions [133]. Thuja, a thujone-rich EO, is abortifacient and contraceptive [122]. Ingestion of thuja oil may cause seizures, convulsions, hypotension, and gastroenteritis [121], and in severe cases, it can cause coma then death [123]. Tansy oil is neurotoxic and carries a risk of causing convulsions [117]. Treatment of female Swiss pregnant mice with methanolic extract of *Artemisia herba-alba* (intragastric gavage at 80 and 150 mg/kg) in the entire period of gestation reduced fertility, altered the physical developments of the offspring, and delayed memory function and neuromotor reflex in the offspring [134]. The aqueous extract of *Artemisia herba-alba* (at 300 mg/kg/day) caused a decrease in fertility ratio of Sprague Dawley female rats [135]. The toxic effects in these studies were attributed to the flavonoid and thujone contents. Based on the available information, consumption of thujone-rich oils should be avoided in pregnancy.

## 6. Apiole-Rich Essential Oils

For many years, parsley (*Petroselinum crispum* (Mill.) Fuss) and its concentrated preparations have been used in South America and Italy to induce abortion, which often ended in death due to severe post abortive vaginal bleeding [136]. The abortifacient effect is attributed to parsley apiole, a main component in most parsley leaf and seed oils. Parsley apiole and dill apiole are bicyclic phenylpropenoid ethers. Parsley apiole is found in parsley seed oil (11.3–67.5%) [58] while dill apiole is found in Indian dill (*Anethum sowa* Roxb. ex Flem.) seed oil (20.7–52.5%) [23] and parsley leaf oil (0.2–5.2%) [22,57]. Parsley apiole poisoning causes severe neurotoxicity which presents a risk of abortion [137]. Signs of parsley apiole intoxication include fever, severe abdominal pain, vaginal bleeding, abortion, convulsions, vomiting, and diarrhea [138]. A single gavage dose of parsley apiole (10 mL/kg) was sufficient to kill all experimental mice within 60 hours due to liver and kidney toxicity [139]. Doses of 5–14 g caused severe hemorrhage and induced abortion in pregnant rabbits [140]. Parsley leaf and seed oils are hepatotoxic, nephrotoxic, and may be abortifacient if taken orally. Topical application of parsley oils is also inadvisable during pregnancy [88]. Since there are no safety thresholds for parsley apiole in humans, internal and external use of parsley apiole-rich essential oils is not recommended in pregnancy due to the high risk of abortion. The structural similarity to parsley apiole suggests that dill apiole could carry the same toxicity and could be hazardous in pregnancy. Therefore, it is best to avoid apiole-rich oils (all routes) throughout pregnancy and breastfeeding [22].

## 7. Camphor-Rich Essential Oils

Camphor is a common component in many essential oils. It is a major component of Ho (*Cinnamomum camphora* (L.) J.Presl) leaf oil (camphor chemotype) (37.8–84.1%) [40], feverfew (*Tanacetum parthenium* (L.) Sch.Bip.) oil (28.0–44.2%) [37], and Spanish lavender (*Lavandula stoechas* L.) oil [64]. Upon consumption, camphor is absorbed immediately via the mucosa and can freely cross the placenta in pregnant women [98] and reach the fetal organs such as brain, liver, lungs, and kidneys [141]. At very high doses, camphor can cause hemorrhage due to severe damage to the placenta [142]. In female mice, camphor (300 mg/kg/day for 20 days) increased the activities of hepatic CYP, glutathione *S*-transferase, and aryl hydrocarbon hydroxylase [143]. Camphor is very toxic to humans with a lethal dose of 5–20 g [144] and 50–550 mg/kg [98]. Camphor can cause damage to several organs including liver, kidney, and brain [145,146]. It can also cause convulsions [147] and induce seizures [148]. Signs of camphor poisoning include seizures, lack of coordination, respiratory depression, nausea, vomiting, and coma [125,149,150]. Despite being neurotoxic, hepatotoxic, and lethal in high doses, camphor is unexpectedly non-teratogenic and non-embryotoxic. In almost fatal doses, it can be reproductively toxic and abortifacient because the fetus lacks the necessary enzymes to metabolize it [151]. Camphor caused a dose-dependent maternal toxicity when given orally to pregnant rats (0.216, 0.464, or 1 g/kg/day on gestational days 6–17) and pregnant rabbits (0.147, 0.316, or 0.681 g/kg/day on gestational days 6–18) [152]. Yet, since camphor is believed to be more toxic to humans than animals, camphor-rich oils should be avoided in pregnancy and breastfeeding.

## 8. Citral-Rich Essential Oils

Citral, 3,7-dimethyl-2,6-octadien-1-al, is a mixture of two geometric isomers, geranial (citral a) and neral (citral b). Citral is a major component in a variety of essential oils including lemon myrtle (*Backhousia citriodora* F. Muell.) (<90.0%) [42], East Indian lemongrass (*Cymbopogon flexuosus* (Nees ex Steud.) W. Watson) (83.0–90%) [41,44,45], West Indian lemongrass (*Cymbopogon citratus* (DC.) Stapf) (77.0–90%) [41,44,45], lemon-scented tea tree (*Leptospermum petersonii* F.M. Bailey) (77%) [46], may chang (*Litsea cubeba* (Lour.) Pers.) (74–78%) [41,48], lemon verbena (*Aloysia triphylla* L’Hérit) (68.0%) [67], honey myrtle (*Melaleuca teretifolia* Endl.) (66.5%) [22], Melissa (*Melissa officinalis* L.) (64.4%) [37,48,153], lemon (*Citrus limon* (L.) Osbeck) leaf (50.0%) [32], lemon basil (*Ocimum* × *africanum* Lour.) (42.2%) [22], Australian lemon balm (*Eucalyptus staigeriana* F. v. Muell. ex F.M. Bailey) (17.6%) [22], and lemon thyme (*Thymus* × *citriodorus* (Pers.) Schreb.) (16.3%) [43]. Citral has a GRAS status and has been added as a flavoring and scenting agent to foods, cosmetics, and various household products (such as detergents, soaps, air fresheners, and insect repellents) to give a lemon or verbena scent. It is also an intermediate for ionone, methylionone, and vitamin A syntheses [154].

Citral is not mutagenic or carcinogenic [155]. However, it has shown some reproductive toxicity in animal studies. Citral reduced the fertility of female Wistar rats through decreasing the number of normal ovarian follicles [156]. Citral is a well-known retinoic acid synthesis inhibitor [157,158,159,160]. In epithelial tissues, citral has been shown to antagonize the activity of vitamin A and prevent the oxidation of retinol to retinoic acid [161]. In mouse epidermis, citral inhibited tissue morphogenesis and tumor production [159,162]. When tested on embryos of white Leghorn chicken, citral showed a dose-dependent teratogenic effect represented by inducing malformations and abnormal eye development [163,164,165]. Citral was reported to act via suppressing the activity of the enzyme ALDH1A1 responsible for retinoic acid synthesis, which in turn affects fetal development [166]. Citral (55 mM) partially inhibited the initiation of meiotic division in human fetal ovary tissues which relies partially on retinoic acid [166]. The oral NOAEL for citral-induced prenatal toxicity was set as < 60 mg/kg/day [167]. Orally administered citral (at 60, 125, 250, 500, and 1000 mg/kg on days 6 to 15 of pregnancy) produced signs of embryo-fetotoxicity (growth retardation, skeletal abnormalities, and spleen weight increase) and maternal toxicity (decreased maternal weight gain, increased resorptions, and impaired implantation) in pregnant Wistar rats [167]. Citral was teratogenic in studies with chick embryos [168,169]. The teratogenic effects of citral were also observed in *Xenopus* embryos treated with 60 mM [170]. After exposure to 1.75 mM of citral for 24 h, tooth development was completely inhibited in 70% CD-1 Swiss mouse embryonic mandible explants while the addition of retinoic acid restored odontogenesis [171]. When injected intra-abdominally into pregnant BALB/c mice (>35 mmol/g on the 9th gestational day), citral caused fetal cranial chondrogenesis and osteogenesis restrictions that diminished by adulthood [158]. However, when given to pregnant Wistar rats (by gavage at 125, 250, 500, or 1000 mg/kg on gestational days 6–15), citral caused maternal toxicity, a dose-dependent increase in resorptions per implantation, and a slight teratogenicity [167]. The mechanism of action seems to involve competing with estrogen for estrogen receptors [172]. When applied directly to the rat’s vagina, citral showed estrogenic effects and caused vaginal hyperplasia [172]. Inhalation of citral (for 6 hr/day on gestation days 6–15 at 10 or 34 ppm as vapor, or 68 ppm as an aerosol/vapor mixture) did not cause teratogenicity in Sprague-Dawley rats [168]. Due to their high citral content, Australian lemon balm, honey myrtle, lemon basil, lemon petitgrain, lemon myrtle, lemon thyme, lemongrass, lemon tea tree, May chang, Melissa, and lemon verbena EOs are teratogenic and their internal use should be restricted during pregnancy [153].

## 9. β-Pulegone-Rich Essential Oils

β-Pulegone (*p*-menth-4(8)-en-3-one) is a monocyclic monoterpenoid ketone found in European pennyroyal (*Mentha pulegium* L.) (67.6–86.7%) [23], North American pennyroyal (*Hedeoma pulegioides* (L.) Pers.) (61.3–82.3%) [23], lesser calamint (*Calamintha nepeta* (L.) Savi) (17.6–76.1%) [47], buchu (*Agathosma betulina* (P.J. Bergius) Pillans) (pulegone chemotype) (31.6–73.2%) [29,30], Turkish pennyroyal (*Micromeria fruticosa* (L.) Druce) (66.7%) [23], and buchu (*Agathosma crenulata* L.) (diosphenol chemotype) (0.6–4.5%) [29]. Following oral consumption, pulegone is metabolized into at least 14 metabolites including menthofuran and 8-pulegone aldehyde which are more toxic than pulegone itself [173,174,175]. Pulegone metabolites are the ones responsible for the toxicity since menthofuran is hepatotoxic and *p*-cresol is a toxin and glutathione-depleting agent [176,177]. γ-Ketoenal, a reactive metabolite of pulegone, causes hepatic injury via covalently binding to cellular proteins in the liver [176,177]. Treatment with cytochrome P-450 inhibitors (SKF-525A, metyrapone, piperonyl butoxide, and carbon disulfide) blocked pulegone hepatotoxicity, indicating the involvement of cytochrome P-450 in pulegone metabolism [175,178]. Unlike menthofuran, (1*R*)-(+)-β-pulegone was reported to deplete hepatic glutathione in in vitro experiments, and upon i.p. injection into rats (at 150 mg/kg) or mice (at 300 mg/kg) [173,179]. In rats, (1*R*)-(+)-β-pulegone (i.p., at 300 mg/kg) caused severe damage to the endoplasmic reticulum that led to cell death [180]. (1*R*)-(+)-β-Pulegone treatment was found to destroy hepatic cytochrome P-450 enzymes [178,180,181,182].

For a long time, pennyroyal has been used as an abortifacient even with its potentially deadly hepatotoxic effects [183]. Pennyroyal oil is hepatotoxic and neurotoxic due to the high content of (6*R*)-(+)-menthofuran and (1*R*)-(+)-β-pulegone [179]. Both *Mentha pulegium* oil and pulegone prevented rat uterine muscle contraction [8]. Pennyroyal intoxication causes severe liver damage, internal hemorrhage, and pulmonary edema [184,185]. Intraperitoneal administration of both pennyroyal oil and pulegone showed similar effects in mice [179]. Since there is no significant medicinal benefit from using β-pulegone-rich oils and due to their hepatotoxicity and the potential of causing abortion, it is best to avoid them in pregnancy and breastfeeding [186].

## 10. Costunolide and Dehydrocostus Lactone-Rich Essential Oils

Costunolide or costus lactone is a bicyclic sesquiterpenoid polyalkene lactone found in costus (*Saussurea costus* (Falc.) Lipsch.) oil (11.0%) [35]. Costunolide is classified as a skin sensitizer (allergen) [187]. Costunolide (i.p., at 100 mg/kg/day) showed anti-angiogenic activity through decreasing vascular endothelial growth factor (VEGF)-induced neovascularization in mice. It also suppressed umbilical vein endothelial cell proliferation in vitro (IC_50_ = 3.4 mM) [188]. Dehydrocostus lactone (DHC) is a sesquiterpenoid lactone found in costus (6.0%). Similar to costus lactone, dehydrocostus lactone is a skin sensitizer [187]. Dehydrocostus lactone exerts its anti-angiogenic action via causing G_0_/G_1_ cell cycle arrest due to the suppression of the Akt/glycogen synthase kinase-3β (GSK-3β)/cyclin D1 and mTOR signaling pathway [189]. Dehydrocostus lactone inhibited angiogenesis in vitro and in mice in a dose-dependent manner [189]. Costus oil is a good example for costunolide and dehydrocostus lactone-rich essential oils [35]. Costus EO is fetotoxic due to its costunolide and dehydrocostus lactone content. Due to the strong anti-angiogenic activity of costunolide and dehydrocostus lactones [188,189] and because of the possible link between anti-angiogenic activity and reproductive toxicity [13,14], it is best to avoid the oil (by any route) during pregnancy and lactation.

## 11. Thymoquinone-Rich Essential Oils

Thymoquinone is a bicyclic benzenoid ketone found in black seed (*Nigella sativa* L.) oil (26.8–54.8%) [27]. It showed reproductive toxicity with an i.p. NOAEL of 15 mg/kg. When administered daily to rats (i.p., at 8 mg/kg), thymoquinone killed most of the animals within a week and the surviving animals had severe peritonitis [190]. It suppressed VEGF-induced angiogenesis in the matrigel plug assay. Subcutaneous administration of thymoquinone to mice (at 6 mg/kg for 15 days) abolished angiogenesis in prostate cancer tumors [191]. Administration of a single dose of thymoquinone (i.p., at 35 or 50 mg/kg on gestational days 11 or 14) to pregnant rats caused a dose-dependent fetal resorption and maternal toxicity [192]. Thymoquinone (by gavage at 10 mg/kg/day on gestational days 1–19) reduced malondialdehyde formation and increased hepatic glutathione in mice with induced gestational diabetes [193]. Due to its strong anti-angiogenic activity [191] and reproductive toxicity, thymoquinone is mostly hazardous in pregnancy. Black seed oil may be fetotoxic because of its high thymoquinone content [191]; therefore, its consumption during pregnancy and breastfeeding should be avoided.

## 12. β-Elemene- and/or β-Eudesmol-Rich Essential Oils

β-Elemene, a monocyclic sesquiterpenoid polyalkene, is one of the major components in atractylis (*Atractylodes lancea* (Thunb.) DC) EO (18.0%) [22], myrrh (*Commiphora myrrha* (Nees) Engl.) EO (8.7%) [50], and katrafay (*Cedrelopsis grevei* Baill. and Courchet) EO (3.0–6.0%) [22]. It is known for its strong anti-angiogenic effects through inhibiting VEGF expression and repressing VEGF-dependent tumor angiogenesis [194,195,196,197,198,199]. Myrrh oil may be fetotoxic based on its β-elemene [174] and furanodiene [200] content and therefore, should be avoided during pregnancy.

β-Eudesmol, a bicyclic sesquiterpenoid alkene alcohol, is a major component in atractylis (*Atractylodes lancea* (Thunb.) DC) (26.0%) [22], araucaria (*Neocallitropsis pancheri* (Carrière) de Laub.) (25.9%) [26], blue cypress (*Callitris intratropica* R.T. Baker and H.B. Sm.) (14.4%) [28], European valerian (*Valeriana officinalis* L.) (0–8.3%), amyris (*Amyris balsamifera* L.) (3.2–7.9%), blue tansy (*Tanacetum annuum* L.) (3.5–6.7%) [117], hinoki leaf (*Chamaecyparis obtusa* (Siebold and Zucc.) Endl.) (6.5%), *Eucalyptus smithii* (6.3%), emerald cypress (*Callitris columellaris* F.Muell.) (5.7%), and vetiver (*Vetiveria zizanioides* (L.) Nash) (0–5.2%) [22]. β-Eudesmol is a known anti-angiogenic agent both in vitro and in vivo via blocking of the extracellular signal-regulated kinase (ERK) signaling pathway [201]. β-Eudesmol showed significant in vitro antiplatelet aggregation activity and best be avoided in childbirth [22]. Based on their β-eudesmol content, atractylis, araucaria, and blue cypress oils may be fetotoxic and may inhibit blood clotting; they should be avoided during pregnancy and lactation.

## 13. Other Essential Oils

(*E*)-Cinnamaldehyde-rich oils, such as cassia (*Cinnamomum cassia* (L.) J. Presl) [23] and cinnamon (*Cinnamomum verum* J. Presl) bark oil [23,24], carry a risk of embryotoxicity and should be avoided during pregnancy and breastfeeding. Cinnamon bark oil has GRAS status, yet it has been shown to lower the number of nuclei and affect the distribution of embryos in pregnant mice (orally, at 375 mg/kg for 2 weeks) [202].

Methyleugenol, a major component in chaste tree (*Vitex agnus-castus* L.) oil, can cause reproductive hormone modulation [33,34]. Moreover, methyleugenol has demonstrated hepatocarcinogenicity in rodents via inducing unscheduled DNA synthesis and forming DNA and protein adducts [89]. It is inadvisable to use methyleugenol-containing oils during pregnancy and breastfeeding. *Vitex agnus-castus* is known to ameliorate premenstrual syndrome (PMS), menstrual cycle irregularity, dysfunctional uterine bleeding, and cyclical breast discomfort [203]. Chaste tree fruit and leaf EOs can alleviate menopausal symptoms [204,205,206]. However, the extract of chaste tree showed dopaminergic activity both in vitro and in vivo via lowering serum estrogen and increasing progesterone levels [204]. Chaste tree fruit and leaf EOs can stimulate dopamine D2 receptors which inhibit prolactin release and normalize the menstrual cycle. Likewise, hyssop (*Hyssopus officinalis* L.) oil (pinocamphone chemotype) is neurotoxic and potentially carcinogenic based on its methyleugenol content [37,41]. Ingestion of hyssop oil might cause epileptiform convulsions [207] and seizures due to its high pinocamphone content [121]. Hyssop oil and *cis-* and *trans-*3-pinanones are GABA_A_ receptor antagonists [208]. Nutmeg (*Myristica fragrans* Houtt.) oil is potentially carcinogenic because of its safrole and methyleugenol content [23,51,89].

Some oil chemotypes like the estragole chemotype of basil (*Ocimum basilicum* L.) oil [23] are toxic based on their estragole content which, in high concentrations, is carcinogenic and should be restricted during pregnancy and lactation [88,89]. Dalmatian sage oil carries a combined risk from its camphor and thujone contents which makes it neurotoxic and embryotoxic [36]. The oil should not be taken orally and its consumption is contraindicated in pregnancy and breastfeeding [88]. Ingestion of dalmatian sage oil can cause convulsions, seizure, coma, and may lead to death [209,210]. Dalmatian sage oil (0.25%, 375 mg/kg for 2 weeks) negatively influenced the distribution of embryos according to nucleus number when fed to pregnant mice [202]. Since the risks of dalmatian sage oil outweigh its benefits, it is best to avoid using it. Hibawood (*Thujopsis dolabrata* (L.f.) Siebold and Zucc.) EO may present a reproductive toxicity because of its β-thujaplicin content [22]. In rats, orally delivered β-thujaplicin caused fetal malformations at 135 mg/kg and a decrease in fetal weight at 45 mg/kg [211]. Nasturtium (*Tropaeolum majus* L.) flower absolute carries a moderate toxicity because of its benzyl cyanide content [22].

In some cases, the hazardous components in the oil have not been identified yet. For instance, carrot (*Daucus carota* L.) seed oil has GRAS status; however, it may interfere with gestation and should be avoided altogether during pregnancy and breastfeeding. It is worth mentioning that the wild carrot plant is reputed as a contraceptive agent. Dong and colleagues have reported that carrot seed oil caused antigestational effects in rats and mice [212]. Subcutaneous injection of carrot seed EO (2.5–5 mL/kg) to female rats and mice inhibited implantation and prevented progesterone synthesis [213]. Another example is oregano (*Origanum vulgare* L.) oil [53,54,55,56]. Although oregano oil has a GRAS status, it is embryotoxic. Orally delivered *Origanum vulgare* EO to pregnant mice (about 150 mg/kg for two weeks) caused an increase in the rate of embryonic cell death [202]. Additionally, zedoary (*Curcuma zedoaria* (Christm.) Roscoe) oil has a GRAS status but its consumption can interfere with gestation and can induce abortion [22]. There was obvious embryotoxicity for zedoary EO ex vivo and reproductive toxicity in animal and developmental experiments [14,174]. In addition, the oil was anti-angiogenic in mice [174], suggesting a strong link between its anti-angiogenic and embryotoxic effects [14]. Chinese zedoary EO (i.p., 300 mg/kg) prevented implantation in a dose-dependent manner in female rats on gestational days 7–9 and prevented about 77% of pregnancies. When administered intra-vaginally to female rabbits at 60 or 400 mg/kg/day on gestational days 5–9 and 2–4, a steam-distilled zedoary EO prevented 16% and 100% of pregnancies, respectively [22]. Aqueous extracts of *C. zedoaria* rhizome (10 g/kg/day for 20 days) demonstrated reproductive toxicity in pregnant mice [214]. The embryotoxic effect of zedoary EO was attributed to its sesquiterpenoids, which can block VEGF-mediated angiogenesis [14]. Nevertheless, there is no direct evidence to link any of the oil components to its antifertility effect. Moreover, zedoary rhizome decoctions and ethanol extracts also have antifertility effects [215].

Rue (*Ruta graveolens* L.) oil may be abortifacient and should be strictly prohibited in pregnancy and breastfeeding. In South America, *Ruta graveolens* ingestion caused abortion [136]. Ingestion of rue aqueous extract was abortifacient, and inhibited implantation in rats at 1 mL/kg [9]. Rue chloroform extract showed antifertility effects due to the presence of chalepensin [9]. Pilocarpine, a compound not present in rue oil, has been suggested as the abortifacient agent [216]. Very little information was found about the toxicity and safety of rue oil. Until further data are available, rue oil should be avoided altogether during pregnancy.

In summary, if essential oil constituents are present in the mother’s circulation, they are expected to reach the fetus and exert some toxic effects. Due to the lack of clinical evidence on reproductive toxicity in humans, it is best to avoid or restrict the use of potentially dangerous essential oil constituents such as anethole, apiole, citral, camphor, thymoquinone, *trans*-sabinyl acetate, methyl salicylate, thujone, pulegone, β-elemene, β-eudesmol, and costus lactone.

## Figures and Tables

**Table 1:** Potentially toxic essential oils and their toxic constituents discussed in this review.

EO	Botanical Name	Family	Part Used	Hazard(s)	Toxic Component(s)	Oil Composition	Maximum Oral Dose in Pregnancy [22]	Ref.
Anise or aniseed	*Pimpinella anisum* L.	Apiaceae	Seeds	Reproductive hormone modulation	(*E*)-Anethole	(*E*)-anethole (75.2–96.1%), *d*-limonene (tr–4.9%), and estragole (0.5–5.0%)	-	[23,24]
Aniseed Myrtle	*Syzygium anisatum* (Vickery) Craven and Biffin	Myrtaceae	Leaves	Reproductive hormone modulation	(*E*)-Anethole	(*E*)-anethole (95.0%), and estragole (4.4%)	-	[25]
Araucaria	*Neocallitropsis pancheri* (Carriere) de Laub. (synonym: *Callitropsis araucarioides* Compton, and *Neocallitropsis araucarioides* (Compton) Florin)	Cupressaceae	Wood	Fetotoxic, anti-angiogenic	β-Eudesmol	β-eudesmol (25.9%), γ-eudesmol (19.0%), α-eudesmol (13.3%), guaiol (6.0%), elemol (5.0%), and β-bisabolenol (4.9%)	-	[26]
Atractylis (Cang-zhu atractylodes)	*Atractylodes lancea* (Thunb.) DC	Asteraceae	Roots	Anti-angiogenic, fetotoxic	β-Elemene and β-eudesmol	β-eudesmol (26.0%), β-elemene (18.0%), hinesol (10.0%), and elemol (6.0%)	-	[22]
Australian Lemon balm (lemon-scented ironbark)	*Eucalyptus staigeriana* F. v. Muell. ex F. M. Bailey	Myrtaceae	Leaves	Teratogenicity	Citral	*d*-limonene+ β-phellandrene (30.5%), geranial (9.9%), neral (7.7%), α-phellandrene (7.1%), and terpinolene (6.6%)	238 mg/day based on 17.6% citral content	[22]
Basil oil (estragole chemotype)	*Ocimum basilicum* L.	Lamiaceae	Leaves	Potentially carcinogenic	Estragole and methyleugenol	estragole (73.4–87.4%), linalool (tr–8.6%), and 1,8-cineole (0.6–6.0%)	-	[23]
Bitter Fennel	*Foeniculum vulgare* Mill. subsp. *capillaceum* Gilib.	Apiaceae	Seeds	Reproductive hormone modulation	(*E*)-anethole	(*E*)-anethole (52.5–84.3%), fenchone (4.0–24.0%), α-pinene (tr-10.4%), *d*-limonene (0.5–9.4%), and estragole (2.8–6.5%).	-	[23]
Black seed (black cumin or black caraway)	*Nigella sativa* L.	Ranunculaceae	Seeds	Fetotoxic	Thymoquinone	thymoquinone (26.8–54.8%), *p*-cymene (14.7–38.0%), longifolene (1.2–10.2%), and α-thujene (1.3–10.1%) as the main constituents.	-	[27]
Blue Cypress (Northern cypress pine)	*Callitris intratropica* R.T. Baker and H.B. Sm.	Cupressaceae	Wood	Fetotoxic, anti-angiogenic	β-Eudesmol	β-eudesmol (14.4%), dihydrocolumellarin (14.0%), guaiol (13.7%), γ-eudesmol (9.1%), α-eudesmol (7.6%), guaiazulene (6.2%), chamazulene (5.6%)	-	[28]
Buchu (diosphenol chemotype)	*Agathosma betulina* Bergius	Rutaceae	Leaves	Abortifacient; hepatotoxicity	Pulegone	isomenthone (4.6–29.1%), limonene (11.6–28.2%), disophenol (12.0–26.3%), menthone (2.5–25.0%), c-diosphenol (10.3–23.3%), and 8-mercapto-*p*-menthan-3-one(*cis-trans*) (0.7–6.6%)	-	[29]
Buchu (pulegone chemotype)	*Agathosma crenulata* L.	Rutaceae	Leaves	abortifacient	Pulegone	(1*R*)-(+)-β-pulegone (31.6–73.2%), isomenthone (3.6–27.6%), limonene (2.1–17.2%), (*E*)-8-acetylthio-*p*-menthan-3-one (0.4–10.4%), and menthone (1.3–7.0%)	-	[29,30]
Carrot seed	*Daucus carota* L. subsp. *sativus* Hoffm.	Apiaceae	Seeds	antigestational effects		carotol (36.1–73.1%), α-pinene (0.9–11.2%), dauca-4,8-diene (1.6–5.9%), and β-caryophyllene (0.7–5.6%)	-	[31]
Cassia (Chinese or false cinnamon)	*Cinnamomum cassia* (L.) J. Presl (synonym: *Cinnamomum aromaticum* Nees)	Lauraceae	Leaves, terminal branches and bark	Embryotoxicity, reproductive toxicity	Methyleugenol and cinnamaldehyde	(*E*)-cinnamaldehyde (73.2–89.4%), (*Z*)-cinnamaldehyde (0.8–12.3%), and (*E*)-cinnamyl acetate (0.1–5.4%) while in the leaf oil (*E*)-cinnamaldehyde (54.6–90.1%), (*E*)-cinnamyl acetate (1.4–12.5%), (*Z*)-cinnamaldehyde (0.4–10.5%), and benzaldehyde (1.1–6.3%)	-	[32]
Chaste tree (Monk’s pepper)	*Vitex agnus-castus* L.	Verbenaceae	Leaves	Reproductive hormone modulation	The oil may contain methyleugenol	Leaf EO: 1,8-cineole (15.6–35.2%), sabinene (6.9–17.1%), α-pinene (1.0–13.9%), α-terpineol (1.4–9.2%), γ-elemene (0–9.1%), β-selinene (0–9.0%), β-caryophyllene (2.3–8.9%), (*Z*)-β-farnesene (0–8.6%), citronellyl acetate (0.3–7.8%), and citronellic acid (0–6.6%). Seed EO: sabinene (7.1–44.1%), 1,8-cineole (8.4–23.3%), α-pinene (1.2–23.1%), γ-elemene (0–17.0%), (*E*)-β-farnesene (0–10.3%), β-caryophyllene (0.8–9.3%), α-terpineol (0.2–9.3%), limonene (0.5–7.4%), (*Z*)-β-farnesene (0–6.9%), citronellyl acetate (0.2–6.0%), β-selinene (0–6.0%), and β-myrcene (0–5.6%).	-	[33,34]
Cinnamon bark	*Cinnamomum verum* J. Presl. (Synonym: *Cinnamomum zeylanicum* Blume)	Lauraceae	Dried inner bark of young trees	Embryotoxicity	(*E*)-Cinnamaldehyde	(*E*)-cinnamaldehyde (63.1–75.7%), eugenol (2.0–13.3%), (*E*)-cinnamyl acetate (0.3–10.6%), linalool (0.2–7.0%), and β-caryophyllene (1.3–5.8%)	-	[23,24]
Costus	*Saussurea costus* (Falc.) Lipsch. (synonym: *Aplotaxis lappa* Decne., *Aucklandia costus* Falc., *Saussurea lappa* (Decne) C.B. Clarke)	Asteraceae	Dried roots	Fetotoxicity, anti-angiogenicity	Costunolide and dehydrocostus lactone	aplotaxene (20.0%), dihydrocostus lactone (15.0%), costusic acid (14.0%), costunolide (11.0%), dehydrocostus lactone (6.0%), and dihydrodehydrocostus lactone (6.0%)	-	[35]
Dalmatian Sage	*Salvia officinalis* L.	Lamiaceae	Leaves	embryotoxic	Camphor, thujones	camphor (7.3–50.2%), α-thujone (13.1–48.5%), borneol (1.5–23.9%), 1,8-cineole (1.8–21.7%), β-thujone (3.9–19.1%), β-caryophyllene (0.2–9.7%), camphene (0–8.6%), α-pinene (0–8.0%) and bornyl acetate (0.3–5.7%)	-	[36]
Feverfew (nosebleed or midsummer daisy)	*Tanacetum parthenium* (L.) Sch. Bip. (synonym: *Chrysanthemum parthenium* (L.) Bernh.)	Asteraceae	Leaves	Unsafe, moderately neurotoxic	Camphor	camphor (28.0–44.2%), (*E*)-chrysanthenyl acetate (22.9–30.2%), camphene (5.4–7.7%), and germacrene D (0.7–4.6%)	-	[37]
Genipi (Genepi)	*Artemisia genepi* Weber. (synonym: *A. spicata* Wulfen, and *A. mutellina* Vill.)	Asteraceae	Aerial parts	Neurotoxic	Thujone	α-thujone (79.8%) and β-thujone (10.4%)	-	[38]
Great Mugwort	*Artemisia arborescens* L.	Asteraceae	Aerial parts	Neurotoxic	Thujone	β-thujone (34.0%), chamazulene (22.4%), and camphor (11.8%)	-	[39]
Green Yarrow (Ligurian yarrow)	*Achillea nobilis* L. (synonym: *A. ligustica* Vis. ex Nym.)	Asteraceae	Aerial parts of the flowering plant	Abortifacient	Sabinyl acetate; camphor	camphor (13.7%) artemisia alcohol (9.2%), germacrene D (8.8%), artemisia ketone (8.7%) and viridiflorol (5.7%)	-	[22]
Ho leaf (camphor chemotype)	*Cinnamomum camphora* (L.) J.Presl	Lauraceae	Leaves	Neurotoxic	Camphor	camphor (37.8–84.1%), 1,8-cineole (1.0–12.0%), and terpinen-4-ol (0.9–6.3%)	-	[40]
Honey Myrtle (Marsh honey myrtle)	*Melaleuca teretifolia* Endl.	Myrtaceae	Leaves	Teratogenicity	Citral	geranial (37.5%), neral (29.0%) and β-myrcene (10.9%)	63 mg/day based on 66.5% citral content	[22]
Hyssop (pinocamphone chemotype)	*Hyssopus officinalis* L.	Lamiaceae	Leaves and flowering tops	Neurotoxicity; carcinogen	Pinocamphone, methyleugenol	pinocamphone (31.2–42.7%), isopinocamphone (30.9–39.2%) and β-pinene (4.0–8.8%)	-	[37,41]
Indian dill seed (Sowa)	*Anethum sowa* Roxb. ex Flem.	Apiaceae	Seeds	hepatotoxic, nephrotoxic, Abortifacient	Dill apiole	dill apiole (20.7–52.5%), d-limonene (5.9–45.0%), (+)-carvone (17.4–23.1%), (*E*)-dihydrocarvone (4.2–16.6%), α-phellandrene (tr–6.5%), and (*Z*)-dihydrocarvone (0.8–5.2%)	-	[32]
Lanyana (African wormwood)	*Artemisia afra* Jacq. ex Willd.	Asteraceae	Leaves and stems	neurotoxic	Thujone	α-thujone (22.5%), (*E*)-chrysanthenyl acetate (19.2%), 1,8-cineole (19.1%), camphor (11.0%), and β-thujone (8.9%)	-	[22]
Lemon basil	*Ocimum* × *africanum* Lour.	Lamiaceae	Leaves	Teratogenicity	Citral	geranial (23.3–25.1%), neral (16.0–17.1%), nerol (13.0–15.3%), linalool (5.0–7.8%), and (*E*)-α-bisabolene (5.3–6.2%)	99 mg/day based on 42.2% citral content	[22]
Lemon leaf (lemon petitgrain)	*Citrus* × *limon* L. (synonym: *Citrus limonum* Risso)	Rutaceae	Leaves	Teratogenicity	Citral	geranial (10.9–39.0%), limonene (8.1–30.7%), neral (6.5–25.3%), geraniol (0.5–15.0%), β-pinene (3.5–13.6%), neryl acetate (3.7–7.4%), nerol (1.3–7.4%), α-terpinyl acetate (tr–7.3%), and linalyl acetate (tr–6.5%)	84 mg based on 50% citral content	[32]
Lemon Myrtle (lemon ironwood or sweet verbena tree)	*Backhousia citriodora* F. Muell.	Myrtaceae	Leaves	Teratogenicity	Citral	geranial (46.1–60.7%) and neral (32.0–40.9%)	46 mg/day	[42]
Lemon Thyme	*Thymus citriodorus* (Pers.) Schreb. (Synonyms: *Thymus lanuginosus* Mill. var. *citriodorum* Pers., *Thymus serpyllum* var. *citriodorus* (Hort.), *Thymus serpyllum* L. var. *vulgaris* Benth.); a cross between *Thymus vulgaris* and *Thymus pulegioides*.	Lamiaceae	Aerial parts	Teratogenicity	Citral	geraniol (39.2%), carvacrol (15.4%), geranial (9.2%) and neral (7.1%)	258 mg/day based on 16.3% citral content	[43]
Lemongrass	*Cymbopogon flexuosus* Nees ex Steud. (synonym.: *Andropogon flexuosus* Nees ex Steud.) (East Indian) and *Cymbopogon citratus* DC (synonym: *Andropogon citratus* DC) (West Indian)	Poaceae	Leaves	Teratogenicity	Citral	East Indian lemongrass: geranial (45.1–54.5%) and neral (30.1–36.1%). West Indian lemongrass: geranial (36.7–55.9%), neral (25.0–35.2%), β-myrcene (5.6–19.2%), geraniol (0–6.7%), and limonene oxide (0–6.4%)	46 mg/day based on 90% citral content	[41,44,45]
Lemon-scented tea tree (lemon tea tree)	*Leptospermum petersonii* F. M. Bailey (synonym: *Leptospermum citratum* Chall., Cheel and Penf.; *Leptospermum liversidgei* R.T. Baker and H. G. Smith)	Myrtaceae	Aerial parts	Teratogenicity	Citral	geranial (45.4%), neral (31.3%), α-pinene (12.3%), and citronellal (6.8%)	54 mg/day based on 77% citral content	[46]
Lesser Calamint (Cuckoo flower, field balm, and nepitella)	*Calamintha nepeta* L. subsp. *glandulosa* Req. (synonym: *Calamintha officinalis* Moench.)	Lamiaceae	Aerial parts	Abortifacient; hepatotoxicity	Pulegone	(1*R*)-(+)-β-pulegone (17.6–76.1%), menthone (7.0–55.8%), piperitone oxide (0–12.4%), piperitone (0–7.4%), piperitenone (0–7.3%), limonene (0.6–7.2%), and terpinen-4-ol (0–6.8%).	-	[47]
May chang (Pheasant pepper tree)	*Litsea cubeba* (Lour.) Pers. (synonyms: *Litsea citrata* Blume, *Laurus cubeba* Lour.)	Lauraceae	Fruits	Teratogenicity	Citral	geranial (37.9–40.6%), neral (25.5–33.8%), limonene (8.4–22.6%), and methyl heptenone (0.5–4.4%)	56 mg/day based on 74% citral content	[41,48]
Melissa (lemon balm)	*Melissa officinalis* L.	Lamiaceae	Fresh aerial parts	Teratogenicity	Citral	geranial (12.5–38.3%), neral (9.7–26.1%), β-caryophyllene (0.3–19.1%), citronellal (4.5–13.3%), germacrene D (0–13.0%), caryophyllene oxide (0.8–10.0%), and geraniol (1.0–8.1%)	65 mg/day based on 64% citral content	[37,48]
Mugwort (chrysanthenyl acetate CT)	*Artemisia vulgaris* L.	Asteraceae	Aerial parts	slightly neurotoxic	Thujone	chrysanthenyl acetate (31.7–32.8%) and germacrene D (12.1–15.9%)	-	[22]
Mugwort or Indian wormwood oil (camphor/thujone CT)	*Artemisia vulgaris* L.	Asteraceae	Aerial parts of flowering plant	Slightly neurotoxic	Thujone	camphor (20.8%), artemisia alcohol (15.3%), α-thujone (11.4%), β-caryophyllene (10.6%), isoborneol (9.3%), 1,8-cineole (9.0%), and sabinene (6.1%)	-	[49]
Myrrh (Somalian myrrh)	*Commiphora myrrha* (Nees) Engl. (synonym: *Commiphora molmol* Engl.)	Burseraceae	Dried gum oleoresin	Fetotoxic, anti-angiogenic	β-Elemene and furanodiene	furanoeudesma-1,3-diene (34.0%), furanodiene (19.7%), lindestrene (12.0%), and β-elemene (8.7%)	-	[50]
Nasturtium (Indian cress) absolute	*Tropaeolum majus* L.	Tropaeolaceae	Flowers	fetal toxicity	Benzyl cyanide, benzyl isothiocyanate	benzyl isothiocyanate (72.3%), unidentified nitrogen compound (16.0%), and benzyl cyanide (2.0%)	-	[22]
Nutmeg	*Myristica fragrans* Houtt (Synonyms: *Myristica officinalis* L. fil., *Myristica moschata* Thunb., *Myristica aromatica* O. Schwartz, and *Myristica amboinensis* Gand.)	Myristicaceae	Kernels	Potentially carcinogenic; reduced fertility	Safrole, methyleugenol, myristicin	East Indian EO: sabinene (14.0–44.1%), α-pinene (18.0–26.5%), β-pinene (8.7–17.7%), myristicin (3.3–13.5%), terpinen-4-ol (1.0–10.9%), γ-terpinene (1.3–7.7%), linalool (0.2–7.4%), limonene (2.0–7.0%), α-phellandrene (0.4–5.8%) and α-terpinene (0.1–5.2%). West Indian EO: sabinene (42.0–57.0%), α-pinene (1.6–12.6%), β-pinene (7.8–12.1%) and terpinen-4-ol (3.0–6.4%)	-	[32,51]
Orange Champaca (golden champa, champak) absolute	*Michelia champaca* L.	Magnoliaceae	Flowers	Toxic	2-Phenylethanol	2-phenylethanol (25.0–34.0%), methyl linoleate (10.0–18.0%), indole (2.9–12.0%), methyl anthranilate (2.1–9.0%), and methyl benzoate (1.0–5.0%)	-	[52]
Oregano	*Origanum onites* L. (synonym: *Origanum smyrnaeum* L.); *Origanum vulgare* L. subsp. *hirtum* (Link) Ietswaart (synonym: *Origanum compactum*, *Origanum hirtum* Link); and *Thymbra capitata* (L.) Cav. (synonym: *Thymus capitatus* L., *Coridothymus capitatus* L., *Satureja capitata* L.)	Lamiaceae	Dried aerial parts of flowering plant	embryotoxic	Not identified	Turkish *Origanum onites* EO: carvacrol (66.5–80.4%), *p*-cymene (3.0–10.9%), and γ-terpinene (1.6–8.7%). Greek/Turkish *Origanum vulgare* subsp. *hirtum* EO: carvacrol (61.6–83.4%), *p*-cymene (4.9–9.7%), and γ-terpinene (3.8–8.2%). Greek *Thymbra capitata* EO: carvacrol (81.5–82.3%) and *p*-cymene (5.8–6.4%).	-	[53,54,55,56]
Parsley leaf	*Petroselinum crispum* Mill (synonym: *P. sativum* Hoffm., and *P. hortenseauct*)	Apiaceae	Leaves	abortifacient	Parsley apiole	Egyptian parsley: *p*-mentha-1,3,8-triene (6.2–45.2%), β-myrcene (7.8–23.8%), β-phellandrene (6.7–19.5%), myristicin (1.9–8.8%), α-pinene (6.9–7.6%), terpinolene (2.8–6.6%), limonene (3.3–5.4%), α-*p*-dimethylstyrene (2.7–5.4%), and dill apiole (0.2–5.2%)	-	[22,57]
Parsley seed	*Petroselinum crispum* Mill	Apiaceae	Seeds	abortifacient	Parsley apiole	parsley apiole (11.3–67.5%), myristicin (0.7–37.9%), allyltetramethoxybenzene (0.6–29.0%), α-pinene (8.3–16.9%), β-pinene (5.4–10.7%), and elemicin (0–8.8%)	-	[58]
Pennyroyal	*Hedeoma pulegioides* L. (N. American); *Mentha pulegium* L. (European) and *Micromeria fruticosa* L. (Turkish)	Lamiaceae	Fresh aerial parts	abortifacient	Pulegone	*Hedeoma pulegioides*: (1*R*)-(+)-β-pulegone (61.3–82.3%) and isomenthone (0.8–31.0%). *Mentha pulegium*: (1*R*)-(+)-β-pulegone (67.6–86.7%), menthone (1.5–16.0%) and isomenthone (0.8–8.6%). *Micromeria fruticosa*: (1*R*)-(+)-β-pulegone (66.7%) and isomenthone (11.1%).	-	[23]
Plectranthus	*Plectranthus fruticosus* L’Hérit	Lamiaceae	Leaves	embryotoxic, fetotoxic, teratogenic and abortifacient	Sabinyl acetate	sabinyl acetate (> 60.0%)	-	[59]
Rue	*Ruta graveolens* L. and *Ruta montana* Mill	Rutaceae	Aerial parts	abortifacient	Not identified	Egyptian *R. graveolens* EO: 2-undecanone (49.2%), 2-nonanone (24.7%), and 2-nonyl acetate (6.2%) Italian *R. graveolens* EO: 2-undecanone (46.8%) and 2-nonanone (18.8%) Non-volatiles: angelicin (0.043%), methoxsalen (0.032%), isopimpinellin (0.02%), bergapten (0.018%), and psoralen (0.015%)	-	[60,61,62]
Savin	*Juniperus sabina* L.	Cupressaceae	Leaves and terminal branches	embryo-fetotoxic, abortifacient and hepatotoxic	*trans*-Sabinyl acetate	*trans*-sabinyl acetate (19.1–53.1%), sabinene (18.3–40.8%), and elemol (tr–7.0%)	-	[23]
Sea Wormwood	*Artemisia maritima* L. (synonyms: *Artemisia contra* Willd. ex Spreng., *Artemisia lercheana* Kar. and Kir., *Artemisia salina* Willd., *Seriphidium maritimum* (L.) Poljakov)	Asteraceae	Leaves and flowering tops	neurotoxic	Thujone	α-thujone (63.3%), sabinene (7.8%) and 1,8-cineole (6.5%)	-	[63]
Spanish Lavender (French lavender or maritime lavender)	*Lavandula stoechas* L. ssp. *stoechas*	Lamiaceae	Flowering tops	neurotoxic	Camphor	camphor (16.4–56.2%), (+)-fenchone (14.9–49.1%), 1,8-cineole (3.6–14.5%), α-pinene (3.4–4.5%), and camphene (2.8–5.5%)	-	[64]
Spanish Sage (lavender sage)	*Salvia lavandulifolia* Vahl (synonym: *Salvia hispanorum* Lag)	Lamiaceae	Flowering tops	abortifacient	Sabinyl acetate	Flowering tops oil: 1,8-cineole (12.0–40.3%), camphor (12.9–36.1%), α-terpinyl acetate (0.5–15.5%), linalool (0.2–11.2%), α-pinene (4.7–10.9%), camphene (4.6–10.6%), β-pinene (3.3–7.3%), *(Z)*-sabinyl acetate (0.5–9.0%), borneol (1.5–6.4%), linalyl acetate (0.1–5.8%), and limonene (2.4–5.0%) Aerial parts oil (steam distilled): 1,8-cineole (21.4–33.8%), α-pinene (10.5–17.5%), β-pinene (6.0–17.3%), limonene (5.6–10.4%), camphor (6.1–9.4%), *trans*-caryophyllene (4.0–8.5%), and myrcene (tr-10.0%).	-	[23,65]
Star anise	*Illicium verum* J.D. Hook.	Illiciaceae	Fruits	reproductive hormone modulation	(*E*)-Anethole	(*E*)-anethole (71.2–91.8%), foeniculin (0.5–14.6%), estragole (0.3–6.6%), and *d*-limonene (0.7–5.0%)	-	[23]
Sweet Birch (black birch or southern birch)	*Betula lenta* L.	Betulaceae	Bark	reproductively toxic	Methyl salicylate and ethyl salicylate	methyl salicylate (90.4%) and ethyl salicylate (5.5%)	-	[22]
Sweet Fennel	*Foeniculum vulgare* Mill.	Apiaceae	Seeds	Reproductive hormone modulation	(*E*)-Anethole	(*E*)-anethole (58.1–92.5%), *d*-limonene (0.2–21.0%), fenchone (0.2–8.0%), and estragole (1.1–4.8%)	-	[23,24]
Tansy	*Tanacetum vulgare* L. (synonyms: *Chrysanthemum tanacetum* Karsch, and *Chrysanthemum vulgare* L.)	Asteraceae	Aerial parts	neurotoxic	Thujone	β-thujone (45.2%), artemisia ketone (10.5%), borneol (7.8%), and bornyl acetate (7.7%)	-	[22]
Thuja (cedar leaf, white cedar, eastern white cedar, eastern arborvitae, or swamp cedar)	*Thuja occidentalis* L.	Cupressaceae	Fresh leaves and terminal branches	neurotoxic	Thujone	α-thujone (48.7–51.5%), fenchone (12.2–12.8%) and β-thujone (7.9–9.9%)	-	[22,66]
Verbena (lemon verbena)	*Aloysia triphylla* L’Hérit (Synonyms: *Aloysia citriodora* Ortega ex Pers., *Lippia citriodora* Ortega ex Pers., and *Lippia triphylla* L’Hérit)	Verbenaceae	Leaves	Teratogenicity	Citral	geranial (29.5–38.3%), neral (22.9–29.6%), and limonene (5.7–15.4%)	61 mg/day based on 68% citral content	[67]
Western red cedar (pacific thuja or western arborvitae)	*Thuja plicata* Donn ex D. Don	Cupressaceae	Needles (leaves)	neurotoxic	Thujones	α-thujone (63.5–84.0%), β-thujone (4.9–15.2%), and sabinene (1.1–8.8%)	-	[68]
White Wormwood (armoise or desert wormwood) α-thujone/camphor chemotype	*Artemisia herba-alba* Asso	Asteraceae	Leaves and flowering tops	neurotoxic	Thujones; camphor	camphor (34.0–55.0%), α-thujone (25.7–36.8%), β-thujone (2.0–9.0%), camphene (0.5–9.0%), and 1,8-cineole (1.5–8.0%)	-	[32,41,69]
Wintergreen	*Gaultheria fragrantissima* Wall. and *Gaultheria procumbens* L.	Ericaceae	Leaves	high doses are teratogenic	Methyl salicylate	Nepalese *G. fragrantissima:* methyl salicylate (97.0–99.5%) Chinese *G. procumbens:* methyl salicylate (96.0–99.0%)	-	[22]
Wormwood (Absinthe)	*Artemisia absinthium* L.	Asteraceae	Leaves and flowering tops	embryo-fetotoxicity; abortifacient	Sabinyl acetate; Thujone	β-thujone chemotype: β-thujone (33.1–59.9%), and *trans*-sabinyl acetate (18.1–32.8%) β-thujone/(*Z*)-epoxy-ocimene chemotype: (*Z*)-epoxy-α-ocimene (24.2–28.9%), β-thujone (20.9–21.7%), and chrysanthendiol (5.3–6.6%) (*Z*)-epoxy-ocimene chemotype: (*Z*)-epoxy-α-ocimene (25.7–42.2%), chrysanthenyl acetate (9.9–15.6%), and sabinyl acetate (0.3–7.4%) sabinyl acetate chemotype: sabinyl acetate (31.5%), neryl isovalerate (9.1%), neryl butyrate (7.9%), and chrysanthenyl acetate (5.8%)	-	[23,39]
Zedoary (white turmeric, hidden ginger)	*Curcuma zedoaria* Roscoe	Zingiberaceae	Rhizome	antifertility; embryotoxicity, antigestational and abortifacient	Not identified	epicurzerene (19.0–46.6%), curzerene (10.4%), curdione (7.0–19.6%), curzerenone (22.3–31.6%), debromofiliforminol (31.5%), 1,8-cineole (18.5–40.8%), β-sesquiphellandrene (21.5%), *p*-cymene (18.4%), curcumenene (18.7%), and α-phellandrene (14.9%)	-	[66,70,71,72,73]

## Abbreviations

EO	essential oil
CNS	central nervous system
GSK-3β	glycogen synthase kinase-3β
sc	subcutaneous
PMS	premenstrual syndrome
GRAS	generally recognized as safe
i.p.	intraperitoneal
IC_50_	median inhibitory concentration
NOAEL	no observed adverse effect level
ppm	parts per million
VEGF	vascular endothelial growth factor
GABA_A_	gamma-aminobutyric acid type A
